# Application Analysis of Positive-Pressure Connector in Invasive Blood Pressure Monitoring in Coronary Interventional Therapy

**DOI:** 10.3389/fsurg.2021.676310

**Published:** 2021-06-16

**Authors:** Aili Wang, Junying Liu, Wanzhong Peng, Yang Jiang, Lina Guo, Zesheng Xu

**Affiliations:** Department II of Cardiology, Cangzhou Central Hospital, Cangzhou, China

**Keywords:** positive pressure joint, invasive blood pressure monitoring, coronary interventional therapy, effectiveness, security

## Abstract

**Background:** In order to reduce the risk of invasive blood pressure monitoring and improve the safety and efficiency, this article mainly analyzes the effectiveness and safety of using positive-pressure connector for invasive blood pressure monitoring in patients with coronary artery interventional therapy, so as to improve the invasive blood pressure monitoring method.

**Aim:** To study and analyze the application of positive-pressure connector in invasive blood pressure monitoring in coronary interventional therapy.

**Methods:** From October 2017 to October 2019, a total of 120 patients admitted to Cangzhou Central Hospital, Cangzhou, Hebei, China, for coronary interventional therapy with invasive blood pressure monitoring were selected and divided into a control group and an experimental group by drawing lots with 60 patients in each group. Positive-pressure connector was used for invasive blood pressure detection in the experimental group, and heparin cap connector was used for invasive blood pressure detection in the control group. The effectiveness and safety of blood pressure monitoring in the two groups were compared, and the influence of different joints on invasive blood pressure monitoring was analyzed.

**Results:** The influencing factors of puncture efficiency in the experimental group (6.67%) were significantly lower than those in the control group (30.00%) (*P* < 0.05). There was no significant difference in catheter bending between the experimental group and the control group (*P* > 0.05). The experimental group exhibited a remarkably higher puncture safety rate (93%) compared to the control group (67%) (*P* < 0.05). There was no significant difference in arterial blood pressure between the two groups with different indwelling time (*P* > 0.05). The frequency of extubation and reinsertion in the experimental group was significantly lower than that in the control group (*P* < 0.05). Factors influencing puncture safety in the experimental group were significantly lower than those in the control group (*P* < 0.05).

**Conclusion:** The use of positive-pressure connector for invasive blood pressure monitoring in patients with coronary artery interventional therapy can greatly improve the safety of blood pressure monitoring and reduce the suffering of patients. Therefore, the application of positive-pressure connector in invasive blood pressure monitoring is worthy of promotion and application in clinical practice.

## Introduction

Invasive blood pressure monitoring is widely applied in clinical practice of blood pressure monitoring. It is a method to measure continuous arterial blood pressure by inserting a puncture tube directly into the artery. Invasive blood pressure monitoring is more accurate than non-invasive blood pressure monitoring and is not affected by measurement manipulation and other factors. In addition, this technique features constant and dynamic change and is affected neither by artificial compression, nor cuff width and tightness. Importantly, the measurement is accurate and reliable and can be obtained at any time (1). This method is generally applied to ambulate blood pressure monitoring of critically ill patients (1, 2). Coronary interventional therapy is a common measure for the treatment of coronary heart disease. Invasive blood pressure monitoring can obtain the complete blood pressure changes in patients with coronary artery interventional therapy, making it convenient for the treatment. However, invasive blood pressure monitoring is prone to infection, residual air at the junction, thrombosis, and other risk factors, and thrombosis at the junction is likely to cause embolism when it enters the artery of the patient, thus exacerbating the patient's condition (2). The heparin cap connector is a three-way switch connector, and the pipelines connected with the three-way switch connector lead to the artery end, the pressure sensor end, and the air end, respectively. In this study, the existing positive-pressure connector used in the control group was modified into a four-way valve from a three-way switch valve, and a positive-pressure connector was installed on the newly added pipeline branch. In this way, the syringe head was directly connected to the positive pressure during blood collection, yielding a closed blood sample collection effect. With the compact structure and low cost of modification, it not only can avoid blood infection, but also can make the operation more convenient and safe (3, 4). Therefore, in the present study, the invasive pressure measurement device of the heparin cap connector was selected as the control, with an aim to provide a promising clinical basis. In order to reduce the risk of invasive blood pressure monitoring and improve the safety and efficiency, this article mainly analyzes the effectiveness and safety of using positive-pressure connector for invasive blood pressure monitoring in patients with coronary artery interventional therapy, so as to improve the invasive blood pressure monitoring method.

## Materials and Methods

### General Materials

In this study, 120 patients admitted to our hospital for coronary interventional therapy with invasive blood pressure monitoring from October 2017 to October 2019, Cangzhou Central Hospital, Cangzhou, Hebei, China, were selected and divided into a control group and an experimental group by drawing lots.

**Figure 1:**
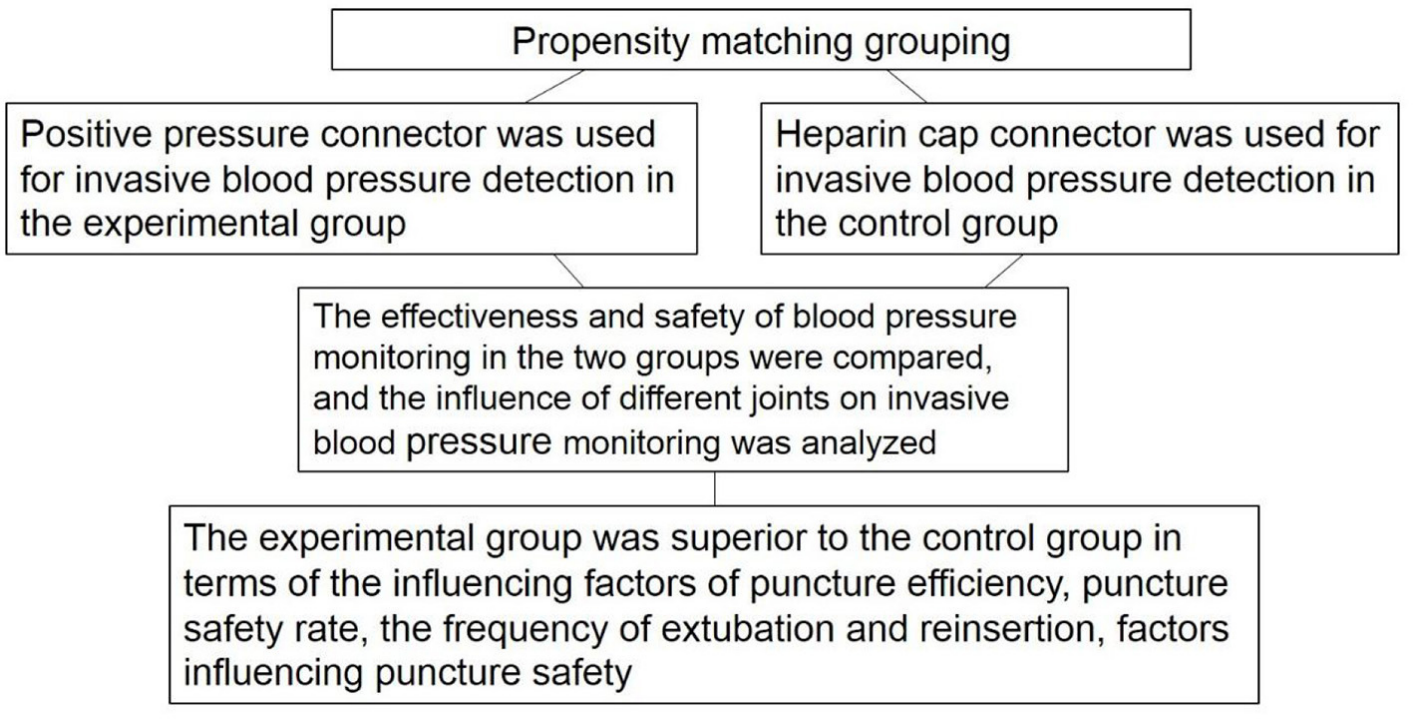
Propensity matching grouping.

The study used propensity score (PS) nearest neighbor matching, the most basic method of propensity score match (PSM), that is, directly looking for one or more individuals with the same or similar PS value from the control group to match the experimental group (Figure 1). There were 60 patients in each group aged 20–78 years. No significant differences were found in age, gender, height, and weight of the patients (*P* > 0.05). See Table 1 for details.

**Table 1:** General information of patients (mean ± SD).

**Group**	**Gender (male/female)**	**Age (years)**	**Body mass index (kg/m^**2**^)**
Experimental group	24/36	57.63 ± 11.71	26.98 ± 7.54
Control group	34/26	59.01 ± 11.22	27.01 ± 6.99
*t*		0.52	0.86
*P*	>0.05	0.60	0.45

### Inclusion Criteria

1. Patients who received coronary interventional therapy and applied invasive blood pressure monitoring in our hospital;
2. No other organic diseases;
3. No drug allergy or abuse history, no bad addiction, normal cognitive function;
4. Did not receive other analgesic drugs recently;
5. Left main trunk diameter stenosis >50%, proximal anterior descending branch diameter stenosis >70%, two or three coronary artery diameter stenosis >70%, and left ventricular function impairment < 40%, extensive ischemia.
6. Any coronary artery diameter stenosis >70%, manifested as activity-induced angina or equivalent symptoms, and poor drug treatment.

### Exclusion Criteria

1. Unstable blood pressure and blood sugar control;
2. Coronary heart disease with other organic diseases;
3. Recent history of infection;
4. The results of Allen test (3) did not support radial artery puncture.

This study was approved by the ethics committee of the hospital, and all patients volunteered to participate in the study and signed informed consent, IRB: 2016-03-423.

In order to ensure the safety and effectiveness of the measurement results, this study has unified management, and there is a relatively complete internal treatment control process, wherein all links are well-considered.

### Methods

#### Blood Pressure Monitoring Method

The starting time is when the puncture was successfully inserted into the pressure measuring tube, and the ending time was when the indwelling needle is removed. In the control group, heparin cap connector was used to connect the pressure sensor with the indwelling needle. Before puncture, it was checked whether each joint of the pressure sensor can be used normally. Alcohol cotton was used to disinfect the front and side of each joint, respectively, and air was exhausted after disinfection to ensure that there was no air residue in the pressure measuring tube. The pressure of the pressurized bag was 300 mm Hg, and heparin saltwater was put into the pressurized bag (4–6). According to the patient's Allen test results, artery was selected for puncture. Taking the radial artery as an example, the middle finger of the left hand was placed at the strongest pulsating point, and the index finger was gently pulled at the distal end to keep the skin appropriately tight. The point 0.5 cm away from the middle finger was selected for the puncture. After the puncture, the needle core was extracted, the trocar was pushed, and the puncture was successful when the blood flow was smooth. The area around the puncture site was sterilized, the needle was fixed with sterile transparent dressing, and the monitor data were calibrated to ensure the pressure sensor was level with the heart.

**Figure 2:**
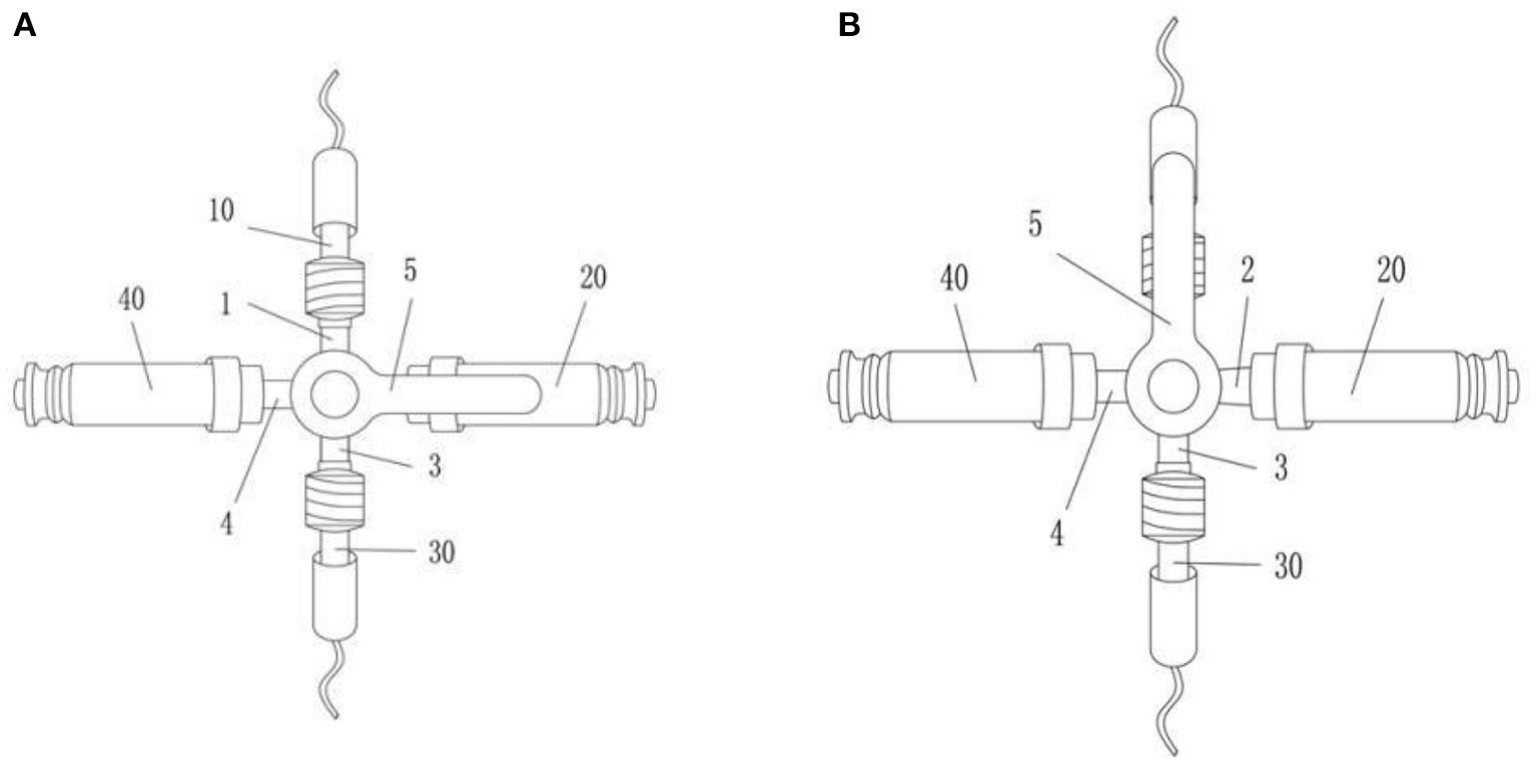
The improved invasive blood pressure monitoring pipeline valve. The improved invasive blood pressure monitoring pipeline valve includes four interconnected pipeline branches (1, 2, 3, 4) and a valve switch (5) that controls the opening and closing of the pipeline branches; the first pipeline branch (1) is connected to the arterial catheter (10), the second pipeline branch (2) is equipped with a pressure sensor screw cap (20), the third pipeline branch (3) is connected with the pressure sensor pipeline (30); the fourth pipeline branch (4) is equipped with a positive-pressure connector (40). When in use, the monitor connected to the pressure sensor needs to be reset to zero, whereas the second pipeline branch (2) is normally closed **(A)**; for zeroing operation, the valve switch is first turned to the first pipeline branch **(B)**; at this time, the first pipeline branch is closed, and the pressure sensor path of the monitor is connected to the air and atmospheric pressure. When the monitor reset button is pressed, and the monitor value shows 0, the valve switch turns to the second pipeline branch **(A)**. At this time, the first pipeline branch, the third pipeline branch, and the fourth pipeline branch are connected, and the second pipeline branch is closed; when blood is collected, the syringe nipple is directly connected to the positive-pressure connector to draw blood.

Heparin cap connector was used to connect the three-way cock on the pressure sensor in the control group, whereas positive-pressure connector was used in the experimental group (Figure 2). Patients' blood pressure and heart rate were monitored. In case of abnormal arterial wave, it was checked whether there was thrombosis in the pressure measuring tube or catheter bending, etc. In case of thrombosis, the thrombus should be pumped back and rinsed in time to avoid intra-arterial embolism (7–10) (Table 2).

**Table 2:** Statistics of drugs and equipment.

**Drug and equipment**	**Specification/batch number**	**Manufacturer**
Heparin	5,000 IU/SFDA certified No. H10980166	Zhaoke Pharmaceutical (Hefei) Co., Ltd.
Arterial cannula	20G SFDA certified 2009 No. 3151394	Hebei Xinlei Medical Equipment Co., Ltd.
Positive-pressure connector	SFDA certified No. 20152641041	Henan Zeyuan Medical Equipment Sales Co., Ltd.
Heparin cap connector	GSM-Z/ SFDA certified 2013 No.3661908	Changyuan Zhongzheng Medical Equipment Sales Co., Ltd.
Pressure sensor	PX260	Shanghai Jumu Medical Equipment Co., Ltd.
Sterile transparent dressing	Shandong FDA certified 2014 No. 1640035	Jining Hanchen Medicine Co., Ltd.

The times of thrombosis and bubbles in the two groups of patients were observed and recorded, and the skin condition of the puncture site was regularly checked. If the patients had red and swollen skin and blood exudation, timely adjustment was made, and if severe tenderness occurred, extubation and reinsertion were conducted.

#### Observation Index

Observation records were made every 5 h to record the effective rate and safety of invasive blood pressure monitoring, and the effective rate refers to the effective rate of puncture and nursing. If blood clots or residual air was discovered at the connector, it was ineffective, and if there was resistance of the syringe pump, it was also ineffective. During the nursing process, if extubation was necessary because of catheter bending thrombosis or that the nursing did not appear in time, the case was considered invalid. The safety index refers to whether the skin at the puncture site is infected; that is, the skin is red, swollen, and prickly, and the blood extravasates, and the wound is seriously purulent. The arterial blood pressure monitoring results of the two groups with different indwelling time were recorded, and the results of the two groups were compared to see whether there was a significant difference.

### Statistical Analysis

The relevant data and data in this article were processed and analyzed by statistical software SPSS version 21.0. Logistic regression was used to score propensity matching. The basic information of the two groups of patients included gender, age, clinical varicose veins, etiology, anatomy, and pathophysiological [Clinical Etiology Anatomic Pathophysiologic Classification System (CEAP)] classification. The CHIVA group and the control group were used as 1/0 binary processing indicators; gender, age, and CEAP classification were used as covariables, and the propensity score matching standard (caliper value) was set to 0.02, and the logistic regression formula was used to perform the propensity matching score with the ratio of 1:1, the propensity score was calculated, and the cases with similar scores were matched. Measurement data were expressed as (mean ± SD) and examined by *t*-test. Enumeration data were expressed as n (%) and examined by *X*^2^ test. *P* < 0.05 was considered statistically significant.

## Results

### Comparison of Influencing Factors of Puncture Efficiency

The puncture effective rate of the control group was lower than that of the experimental group, and the difference was significant (*P* < 0.05). The indwelling time of patients in the experimental group was not statistically different from the control group 5.33 ± 1.02 vs. 5.01 ± 1.74 days, *P* = 0.24). The influencing factors of puncture efficiency in the two groups are shown in Table 3.

**Table 3:** Comparison of influencing factors of puncture efficiency [*n* (%)].

**Group**	**Blocked pipe**	**Air residue in the connector**	**Thrombus at the connector**
Experimental group (*n* = 60)	0 (0)	1 (1.67)	3 (5.00)
Control group (*n* = 60)	5 (8.33)	6 (10.00)	7 (11.67)
*X*^2^	2.64	1.36	4.35
*P*	0.02	0.04	0.03

### Comparison of Influencing Factors of Nursing Efficiency

**Figure 3:**
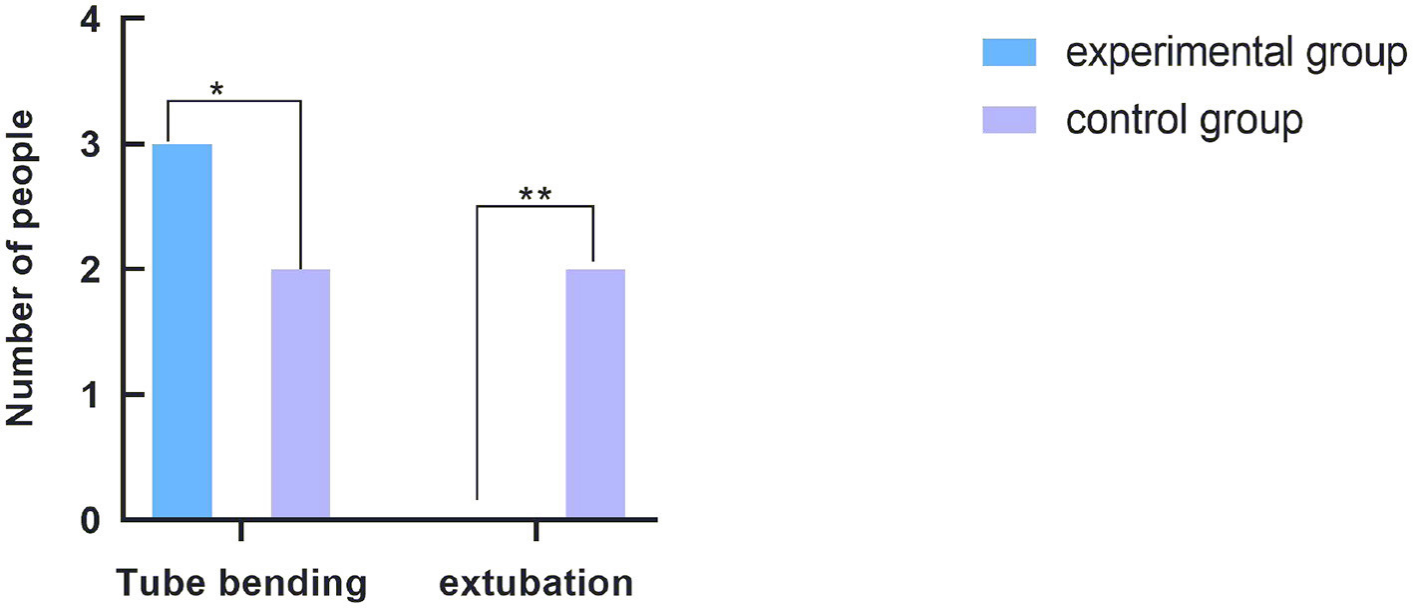
Comparison of influencing factors of nursing efficiency. The abscissa indicates the factors affecting the nursing efficiency. From left to right, the coordinates are tube bending and extubation. The ordinate indicates the number of people. *The comparison of catheter bending in the nursing process between the two groups, with three cases in the experimental group and two cases in the control group. The difference was not statistically significant (*P* = 0.64). **The comparison of extubation and reinsertion in the nursing process between the two groups. There were no cases in the experimental group and two cases in the control group, and the difference was not statistically significant (*P* = 0.15).

The total effective rate of the experimental group was not statistically different from that of the control group (90 vs. 87%, *P* > 0.05). The specific comparison results are shown in Figure 3.

### Comparison of Influencing Factors for Puncture Safety

**Figure 4:**
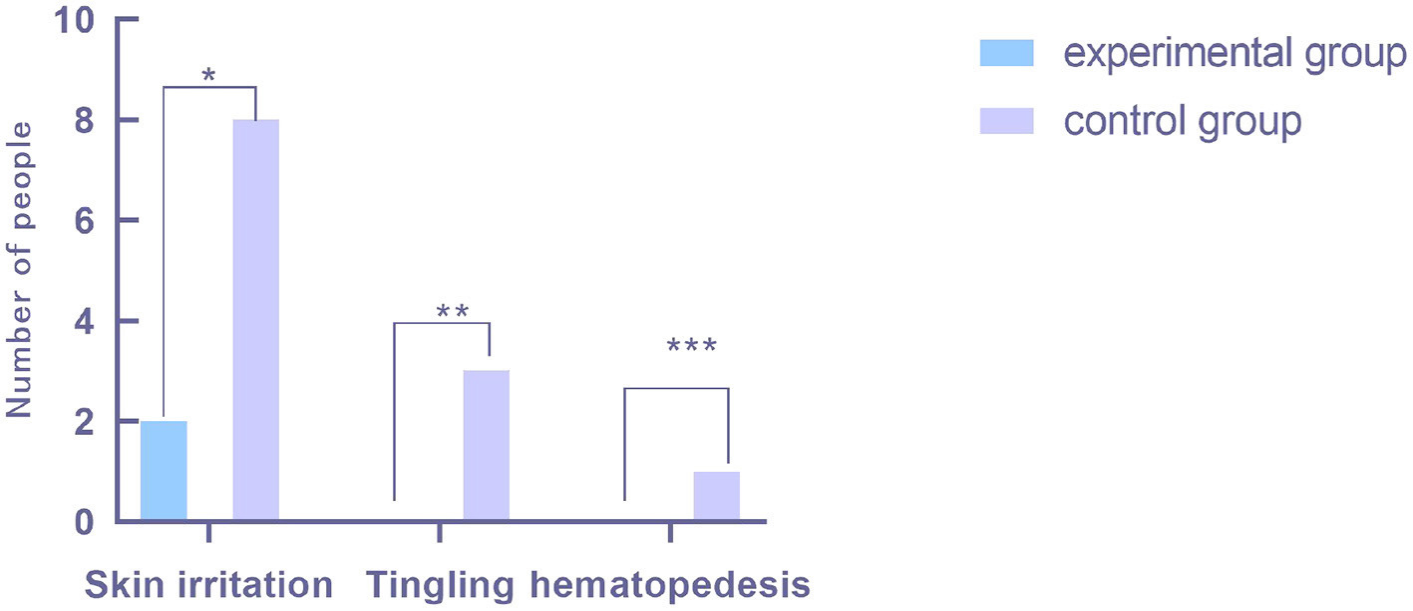
Comparison of security influencing factors. The *x*-coordinate represents the factors affecting puncture safety. From left to right, it is skin redness and swelling, skin tingling, and blood oozing at puncture point. The *y*-coordinate represents the number of people. *There was a significant difference in the number of patients with skin redness and swelling at the puncture site between the two groups, including two patients in the experimental group and eight patients in the control group, and the difference was statistically significant (*P* = 0.04). **The comparison of the number of patients with skin prickling at the puncture site between the two groups. There were no cases in the experimental group and three cases in the control group, and the difference was statistically significant (*P* = 0.07). ***The comparison of the number of patients with blood exudation at the puncture site between the two groups. There were no cases in the experimental group and one case in the control group, and the results were not statistically significant (*P* = 0.31).

Because invasive blood pressure monitoring requires arterial puncture, and heparin water injection needs to flow through the connector into the body, the use of heparin cap connector during this process will inevitably lead to infection. During the monitoring time of blood pressure in the two groups, only two patients in the experimental group had skin redness and swelling, the puncture safety rate being 93%. A total of 12 patients in the control group showed signs of infection such as skin redness, swelling, tingling, and oozing blood, the puncture safety rate being 67%. Comparison of puncture safety between the two groups were statistically significant (*P* = 0.01). The comparison of factors affecting safety, such as puncture wound infection, is shown in Figure 4.

### Comparison of Arterial Blood Pressure Test Results for Different Indwelling Time

**Figure 5:**
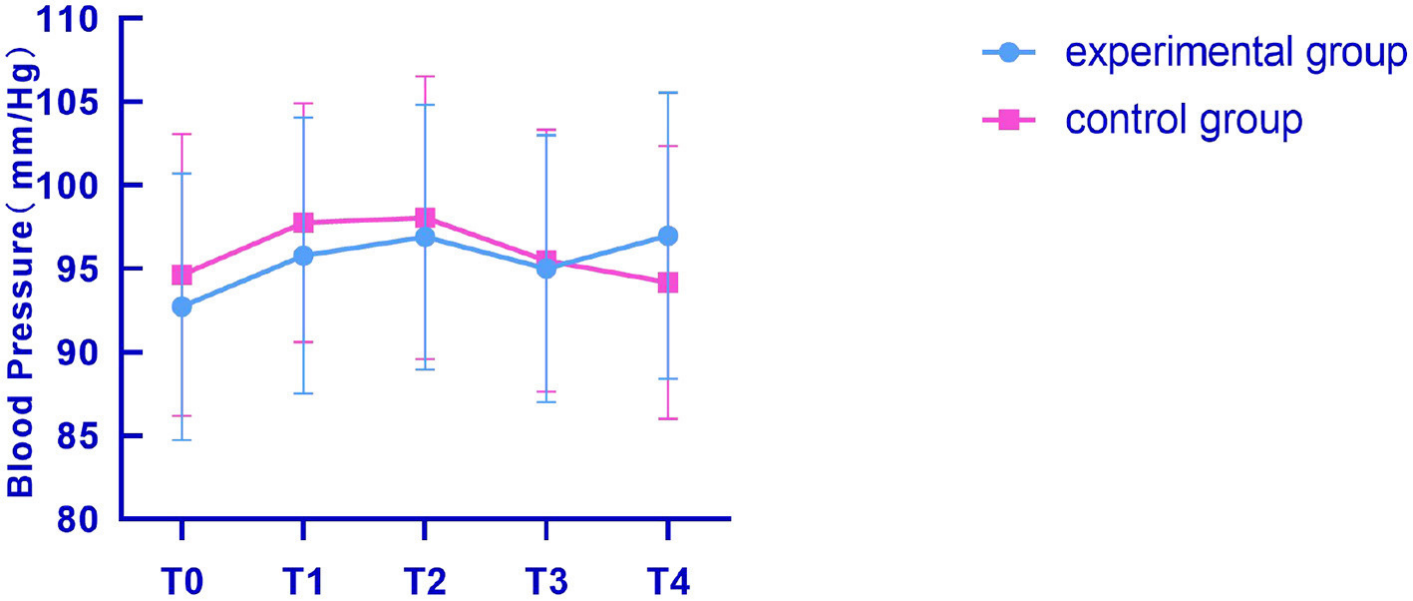
Comparison of arterial blood pressure changes between the two groups at different times. The *x*-coordinates T0, T1, T2, T3, and T4, respectively, represent 1 h, 1, 2, 3, and 4 d after puncture, and the *y*-coordinate represents the arterial blood pressure measured in the two groups of patients. The figure indicated that the blood pressure of the two groups was not significantly different 1 h after puncture. The experimental group was 92.73 ± 7.99, and the control group was 94.63 ± 8.45 (*t* = 0.89, *P* = 0.37). One day after puncture, there was no significant difference in blood pressure between the two groups: 95.79 ± 8.26 in the experimental group and 97.77 ± 7.15 in the control group (*t* = 0.99, *P* = 0.33). There was no significant difference in blood pressure between the two groups 2 d after puncture: 96.91 ± 7.94 in the experimental group and 98.06 ± 8.47 in the control group (*t* = 0.54, *P* = 0.59). There was no significant difference in blood pressure between the two groups 3 d after puncture. The difference was not statistically significant between the experimental group (95.01 ± 8.00) and the control group (95.49 ± 7.84) (*t* = 0.23, *P* = 0.82). There was no significant difference in blood pressure between the two groups 4 d after puncture, indicating that there was no statistical significance between the experimental group (96.98 ± 8.57) and the control group (94.18 ± 8.17) (*t* = 1.29, *P* = 0.20).

The arterial blood pressure monitoring results at 1 h, 1, 2, 3, and 4 d after puncture were recorded and represented by T0, T1, T2, T3, and T4, respectively. The changes of arterial blood pressure in the two groups at different indwelling time were analyzed and compared. The comparison results were *P* > 0.05, showing no statistical significance. The specific comparison results are shown in Figure 5.

## Discussion

Invasive blood pressure monitoring in coronary interventional therapy can accurately obtain patients' arterial blood pressure and timely observe patients' condition changes based on the changes of patients' blood pressure waveform, which is convenient for early detection of possible problems in treatment and timely formulation of treatment countermeasures (11, 12). Invasive blood pressure monitoring refers to inserting a puncture needle directly into the artery, which is not affected by the measurement technique compared with non-invasive blood pressure. Therefore, invasive blood pressure monitoring has been widely used in clinical medical work at present. However, invasive blood pressure monitoring requires the insertion of the puncture needle into the artery and retention for a period of time, which is highly likely to lead to complications such as wound infection at the puncture site of the patient, aggravating the treatment workload. At the same time, thrombosis is easy to form in the catheter, which may form embolism when entering the artery of the patient, affecting the patient's health (12). The positive-pressure connector is a kind of connector that can be kept absolutely sealed, and the connectionless state can guarantee the absolute sterility. In order to reduce the risk of invasive blood pressure monitoring, the use of positive-pressure connector instead of heparin cap connector to connect the indwelling needle with the pressure sensor can reduce the possibility of thrombosis in the connector and greatly reduce the risk of wound infection in patients, thus ensuring the safety of patients (13–15).

According to the results, the effective rate of puncture in the experimental group was significantly higher than that in the control group. In the control group, heparin cap connector was used to connect pressure sensors with indwelling needles, and 21 patients were found to have failed puncture, with the puncture effective rate of 30%, which was significantly different from the 87% in the experimental group. Therefore, the positive-pressure connector can improve the puncture efficiency, reduce the risk of embolization in the artery of patients with thrombosis in the connector, and improve the accuracy of invasive blood pressure monitoring. It is consistent with the results of invasive blood pressure monitoring proposed by some relevant studies (15, 16) that invasive blood pressure monitoring results with a smaller error value are more accurate than non-invasive ones.

In the nursing process, catheter may be bent, resulting in thrombosis and, in severe cases, even extubation and reinsertion (16–18). In this study, the difference of nursing efficiency between the two groups was not significant (*P* = 0.69). However, whether the nursing work has an effect on the efficiency of invasive blood pressure detection needs further study.

Puncture infection is an important factor affecting puncture safety. As invasive blood pressure monitoring requires puncture of the artery, and the injection of heparin water needs to flow through the connector into the body, the use of heparin cap connector during this process will inevitably lead to infection. In this study, two patients in the experimental group had redness and swelling of the puncture site, and the puncture safety was 93%. A total of 12 patients in the control group showed signs of infection such as skin redness, swelling, tingling, etc., and puncture safety was 67%. The comparison of puncture safety between the groups were significantly different (*P* = 0.001).

In addition, the blood pressure waveform and numerical changes in the two groups were recorded, respectively, in the same time period, and no significant difference was found in the blood pressure changes in the two groups in the same time period (*P* > 0.05). The results indicated that the difference between positive-pressure connector and heparin cap connector in invasive blood pressure monitoring lies in the possibility of causing complications, which is not closely related to the accuracy of blood pressure monitoring results. This result is similar to the one proposed by Nunes et al. (2) that the results of invasive blood pressure monitoring are independent of arterial puncture. The defect of the heparin cap three-way switch used in the control group is that the screw cap of the three-way switch needs to be removed when blood samples are collected. Frequent opening of the screw cap of the three-way switch can easily cause blood pollution, occupational exposure of medical staff, and contaminated screw cap with no special screw cap replacement. At the same time, the heparin cap needs to be removed when the zero correction is required, which not only wastes consumables and increases the patient's medical expenses, but also there are still problems such as blood pollution and occupational exposure of medical staff. Therefore, it is necessary to improve the existing invasive blood pressure monitoring pipeline valve to reduce the risk of blood contamination and occupational exposure of medical staff during blood sampling. The positive-pressure device used in the experimental group is improved on the basis of the control group, which can effectively reduce occupational exposure risks, and is conducive to convenient and safe operation for medical staff. The limitation of this study is that this is a single-center study with a small scale. In the future, the study will be expanded, and long-term follow-up trials will be conducted. The stability of the new and modified equipment used in the study needs further exploration. To sum up, the application of invasive blood pressure monitoring in coronary interventional therapy and the use of positive-pressure connector can greatly reduce the risk of treatment and reduce the occurrence of related complications, which is conducive to the recovery and treatment of patients. At the same time, the positive-pressure connector replacing the heparin cap connector can guarantee the absolute seal and reduce the possibility of infection. Therefore, the positive-pressure connector is worthy to be popularized and applied in invasive blood pressure monitoring of coronary interventional therapy.

## Data Availability Statement

The raw data supporting the conclusions of this article will be made available by the authors, without undue reservation.

## Ethics Statement

The studies involving human participants were reviewed and approved by Cangzhou Central Hospital. The patients/participants provided their written informed consent to participate in this study.

## Author Contributions

AW and JL contributed to the study design and reviewed the manuscript. WP and AW analyzed the data and wrote the manuscript. YJ, LG, and ZX contributed to the data collection, data interpretation, and manuscript writing. All authors read and approved the final manuscript.

## Conflict of Interest

The authors declare that the research was conducted in the absence of any commercial or financial relationships that could be construed as a potential conflict of interest.
